# Synthesis and Crystal Structures of *N*-Substituted Pyrazolines

**DOI:** 10.3390/molecules18022386

**Published:** 2013-02-20

**Authors:** Wan-Sin Loh, Ching Kheng Quah, Tze Shyang Chia, Hoong-Kun Fun, Majal Sapnakumari, Badiadka Narayana, Balladka Kunhanna Sarojini

**Affiliations:** 1 X-ray Crystallography Unit, School of Physics, Universiti Sains Malaysia, Penang 11800 USM, Malaysia; 2 Department of Pharmaceutical Chemistry, College of Pharmacy, King Saud University, Riyadh 11451, Saudi Arabia; 3 Department of Studies in Chemistry, Mangalore University, Mangalagangotri-574 199, India; 4 Department of Chemistry, P.A. College of Engineering, Nadupadavu, Mangalore-574 153, India

**Keywords:** synthesis, X-ray diffraction, pyrazole, crystal structure

## Abstract

Four pyrazole compounds, 3-(4-fluorophenyl)-5-phenyl-4,5-dihydro-1*H*-pyrazole-1-carbaldehyde (**1**), 5-(4-bromophenyl)-3-(4-fluorophenyl)-4,5-dihydro-1*H*-pyrazole-1-carbaldehyde (**2**), 1-[5-(4-chlorophenyl)-3-(4-fluorophenyl)-4,5-dihydro-1*H*-pyrazol-1-yl]ethanone (**3**) and 1-[3-(4-fluorophenyl)-5-phenyl-4,5-dihydro-1*H*-pyrazol-1-yl]propan-1-one (**4**), have been prepared by condensing chalcones with hydrazine hydrate in the presence of aliphatic acids, namely formic acid, acetic acid and propionic acid. The structures were characterized by X-ray single crystal structure determination. The dihedral angles formed between the pyrazole and the fluoro-substituted rings are 4.64(7)° in **1**, 5.3(4)° in **2** and 4.89(6)° in **3**. In **4**, the corresponding angles for molecules *A* and molecules *B* are 10.53(10)° and 9.78(10)°, respectively.

## 1. Introduction

The reaction of chalcones with hydrazine derivatives is one of the most extensively applied reactions in organic synthesis. Investigations revealed that in most cases the products formed are pyrazoline derivatives [1,2,3,4]. However, the reactions may also produce pyrazole derivatives [5] and Schiff base hydrazones [6]. These reactions are usually carried out in an acidic medium.

Pyrazoline derivatives display various biological activities such as antitumor, antifungal, antiviral, antiparasitic, anti-inflammatory and analgesic, antimycobacterial, anticancer, antibacterial, insecticidal, antinociceptive, hypotensive, antidepressant, photoluminiscence, anti-tubercular, antiamoebic, MAO-inhibitory, amine oxidase inhibitory and antioxidant properties [7,8,9]. Several 1,3,5-triaryl-2-pyrazoline derivatives were also used as scintillation solutes [10]. Pyrazoline derivatives with a phenyl group at the 5-position have been shown to possess good film-forming properties and exhibit excellent characteristics of blue photoluminescence, fluorescence and electroluminescence [11].

In view of importance of pyrazolines and in continuation of our work on the synthesis and structure determination of various pyrazoline derivatives [12,13,14,15], we report the synthesis and crystal structures of four novel *N*-substituted pyrazolines.

## 2. Results and Discussion

**Scheme 1:**
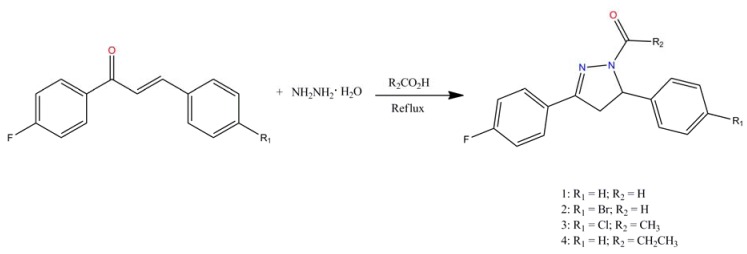
Preparation of *N*-substituted pyrazoline compounds.

One of the most convenient methods for the synthesis of pyrazolines is the reaction of α,β-unsaturated ketones with hydrazine hydrate and its derivatives. New *N*-substituted pyrazolines, **1**–**4** have been synthesized by the reaction of respective chalcone with hydrazine hydrate in the presence of different aliphatic acids as shown in Scheme 1. The crystallographic data for the four compounds are listed in Table 1.

### 2.1. Crystal Structure Description of Compound ***1***

**Figure 1:**
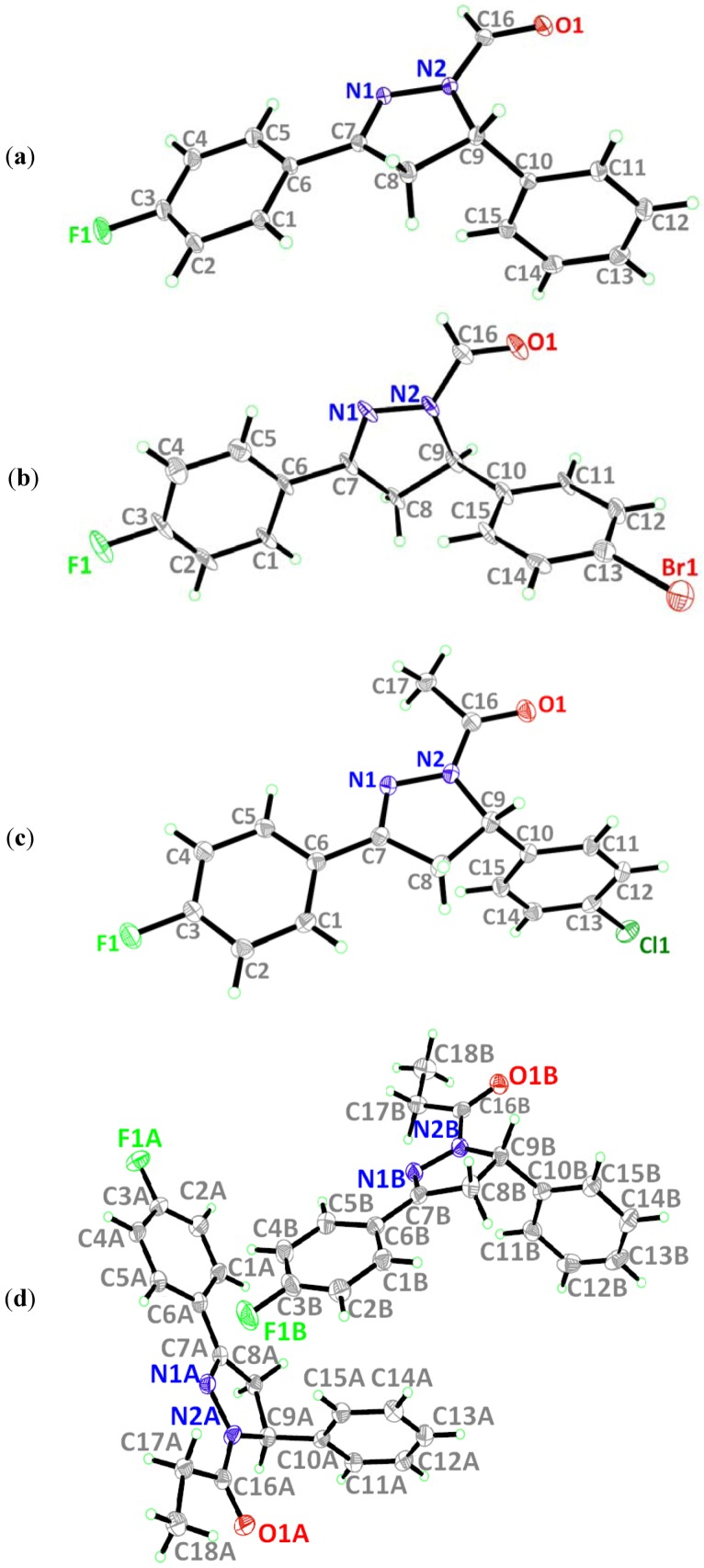
(**a**)–(**d**) ORTEP diagrams of **1**–**4** drawn at 50% ellipsoids for non-hydrogen atoms.

**Figure 2:**
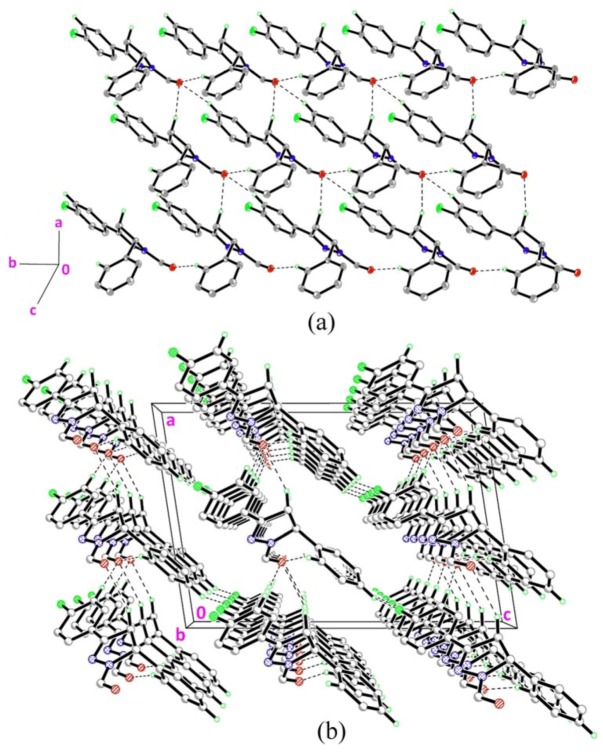
(**a**)–(**b**) Crystal structure of compound **1** with intermolecular hydrogen bonding patterns shown as dashed lines. H atoms not involved in the crystal structure have been omitted for clarity.

The molecular structure of **1** is depicted in Figure 1a. The pyrazole ring (N1/N2/C7–C9) is almost planar with a r.m.s deviation of 0.0457Å. This ring forms a dihedral angle of 4.64(7)° with the fluoro-substituted benzene ring (C1–C6) and is almost perpendicular with the benzene ring (C10–C15), with a dihedral angle of 84.83(7)°. Bond lengths and angles are within the normal ranges [16]. In the crystal structure of **1**, the molecules are linked into planes parallel to the *ab*-plane by intermolecular C–H•••O hydrogen bonds as shown in Figure 2a (Table 2). These planes are further linked into a three-dimensional network by intermolecular C–H•••F hydrogen bonds (Figure 2b; Table 2).

**Table 1:** Crystal data and parameters for structure refinement of **1**, **2**, **3** and **4**.

Compound	1	2	3	4
CCDC deposition numbers	895315	895316	895317	895318
Molecular formula	C_16_H_13_FN_2_O	C_16_H_12_BrFN_2_O	C_17_H_14_ClFN_2_O	C_18_H_17_FN_2_O
Molecular weight	268.28	347.19	316.75	296.34
Crystal system	Monoclinic	Monoclinic	Monoclinic	Triclinic
Space group	*Cc*	*Cc*	*P*2_1_/*c*	*P* 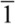
*a*/Å	11.9069(7)	6.2375(14)	6.1087(4)	9.3334(2)
*b*/Å	6.2516(4)	12.191(3)	12.3725(9)	13.2760(3)
*c*/Å	17.3103(10)	18.703(4)	19.6142(14)	13.2857(3)
α/°	90	90	90	107.052(1)
β/°	98.737(1)	93.239(5)	97.769(1)	95.124(1)
γ/°	90	90	90	96.629(1)
*V*/ Å^3^	1273.58(13)	1419.9(6)	1468.83(18)	1550.14(6)
*Z*	4	4	4	4
*D*_calc_ (g cm^−3^)	1.399	1.624	1.432	1.270
Crystal Dimensions (mm)	0.19 × 0.32 × 0.46	0.13 × 0.29 × 0.41	0.17 × 0.17 × 0.29	0.11 × 0.17 × 0.29
μ/mm^−1^	0.10	2.906	0.274	0.088
Radiation λ (Å)	0.71073	0.71073	0.71073	0.71073
*T*_min_/*T*_max_	0.9559/0.9810	0.3792/0.7074	0.9238/0.9549	0.9753/0.9907
Reflections measured	7119	9227	16261	33624
Ranges/indices (*h*, *k*, *l*)	−16, 16; −8, 8; −22, 24	−8, 7; −15, 15; −24, 24	−8, 8; −16, 17; −22, 27	−13, 11; −18, 18; −18, 18
θ limit (°)	2.4-30.1	2.2-27.5	2.0-30.2	1.6-30.1
Unique reflections	1866	2748	4340	9058
Observed reflections (*I* > 2σ(*I*))	1847	2600	3706	5096
Parameters	181	172	200	399
Goodness of fit on *F*^2^	1.07	1.131	1.047	1.043
*R*_1_, *wR*_2_ [*I* ≥ 2σ(*I*)]	0.0267, 0.0748	0.0672, 0.1814	0.0341, 0.1010	0.0628, 0.1538

**Table 2:** Hydrogen bond geometries for compounds (**1**), (**2**), (**3**) and (**4**).

*D*–H···*A*	*d*(*D*–H) (Å)	*d*(H···*A*) (Å)	*d*(*D*···*A*) (Å)	*Angle* (*D*–H···*A*) (°)
(1)				
C2–H2A···O1 ^i^	0.95	2.36	3.2950(16)	166
C8–H8B···O1 ^ii^	0.99	2.60	3.5011(16)	151
C13–H13A···F1 ^iii^	0.95	2.53	3.1809(17)	126
C15–H15A···O1 ^iv^	0.95	2.36	3.2691(17)	161
(2)				
C2–H2A···O1^v^	0.95	2.37	3.305(10)	167
C12–H12A···F1^vi^	0.95	2.48	3.322(10)	148
C15–H15A···O1^vii^	0.95	2.37	3.257(10)	155
(3)				
C2–H2A···O1v^iii^	0.93	2.43	3.2492(14)	147
C14–H14A···F1^ix^	0.93	2.49	3.3462(14)	154
C15–H15A···O1 ^x^	0.93	2.52	3.4282(14)	166
(4)				
C1A–H1AA···O1B ^xi^	0.95	2.43	3.324(2)	156
C1B–H1BA···O1A ^xii^	0.95	2.43	3.223(2)	141
C9B–H9BA···O1A ^xiii^	1.00	2.56	3.345(2)	135
C15B–H15B···O1A ^iv^	0.95	2.48	3.349(2)	151

^i^ 1/2 + *x*, 3/2 + *y*, *z*; ^ii^ 1/2 + *x*, 1/2 + *y*, *z*; ^iii^ −1/2 + *x*, 3/2 − *y*, 1/2 + *z*; ^iv^*x*, 1 + *y*, *z*; ^v^ −3/2 + *x*, −1/2 + *y*, *z*; ^vi^ 3/2 + *x*, 3/2 − *y*, 1/2 + *z*; ^vii^ −1 + *x*, *y*, *z*; ^viii^ 1 − *x*, −1/2 + *y*, 3/2 − *z*; ^ix^ 2 − *x*, 1/2 + *y*, 3/2 − *z*; ^x^ 1 + *x*, *y*, *z*; ^xi^ 1 − *x*,1 − *y*, 1 − *z*; ^xii^ 1 − *x*, −*y*, −*z*.

### 2.2. Crystal Structure Description of Compound ***2***

**Figure 3:**
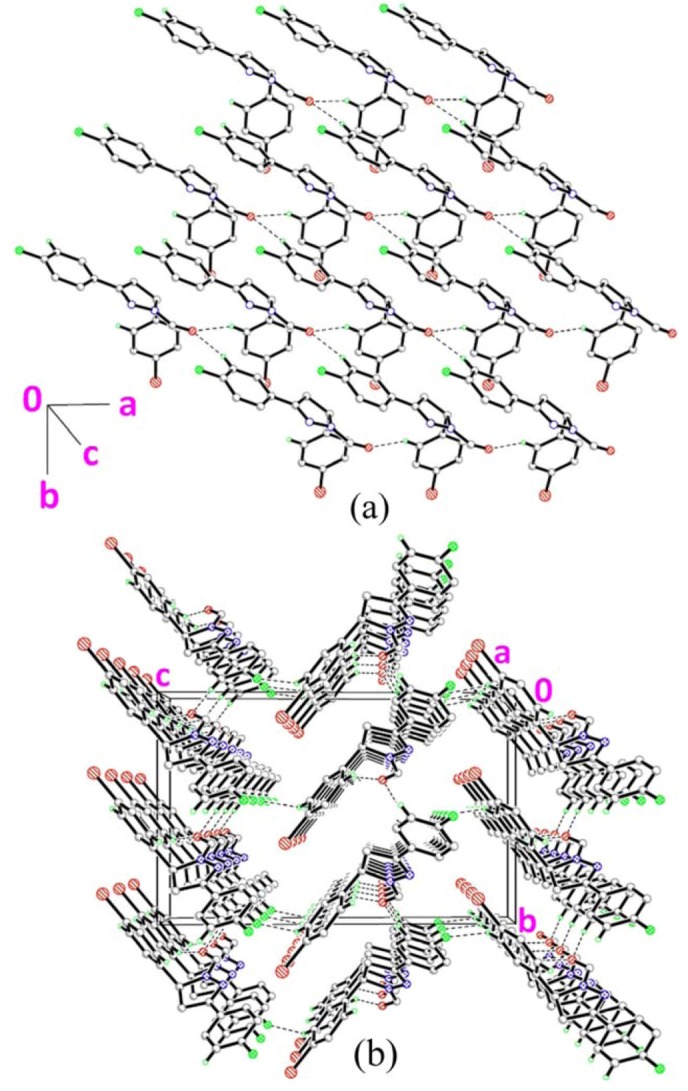
(**a**)–(**b**) Crystal structure of compound **2** with intermolecular hydrogen bonding patterns shown as dashed lines. H atoms not involved in the crystal structure have been omitted for clarity.

Figure 1b shows the molecular structure of **2**. The pyrazole ring (N1/N2/C7–C9) with r.m.s deviation of 0.0434Å is almost coplanar with the fluoro-substituted benzene ring (C1–C6) with a dihedral angle of 5.3(4)° and it is almost perpendicular with the bromo-substituted benzene ring (C10–C15) with a dihedral angle of 85.1(4)° which is identical to that of compound **1**. Bond lengths and angles are within the normal ranges [16]. The crystal structure of **2** is shown in Figure 3. Intermolecular C–H•••O hydrogen bonds (Table 2) link the molecules to form planes parallel to the *ab*-plane as shown in Figure 3a. These planes are further inter-connected into a three-dimensional network via C–H•••F hydrogen bonds (Table 2; Figure 3b).

### 2.3. Crystal Structure Description of Compound ***3***

**Figure 4:**
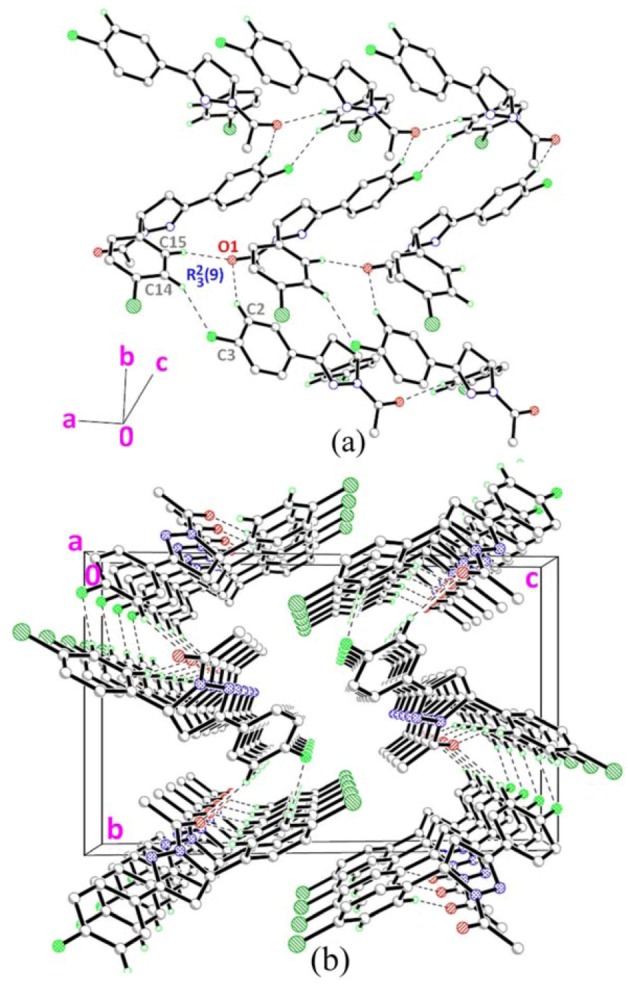
(**a**)–(**b**) Crystal structure of compound **3** with intermolecular hydrogen bonding patterns shown as dashed lines. H atoms not involved in the crystal structure have been omitted for clarity.

The molecular structure of **3** is given in Figure 1c. The pyrazole ring (N1/N2/C7–C9) with r.m.s deviation of 0.0259Å, forms dihedral angles of 4.89(6) and 85.76(6)°, respectively, with the fluoro- and chloro-substituted benzene rings (C1–C6 & C10–C15). Bond lengths and angles are within the normal ranges [16]. Figure 4 shows the crystal structure of **3**. The molecules are linked together via two C–H•••O and one C–H•••F hydrogen bonds (Table 2), generating $R_{3}^{2}$ *R*2 3(9) ring motifs [17] and form planes parallel to the *ab*-plane.

### 2.4. Crystal Structure Description of Compound ***4***

**Figure 5:**
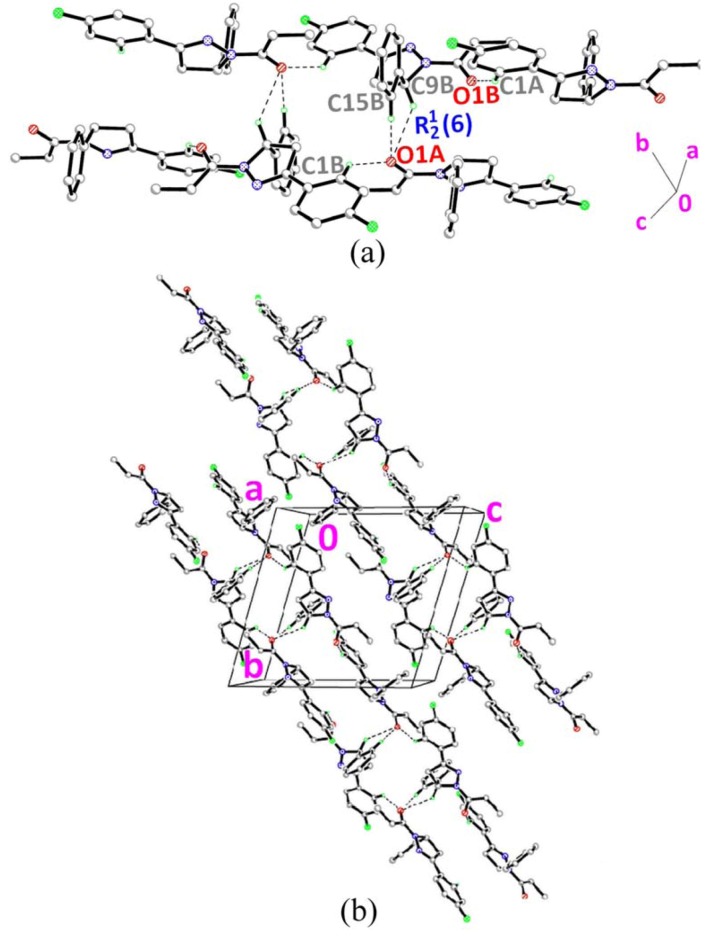
(**a**)–(**b**) Crystal structure of compound **4** with intermolecular hydrogen bonding patterns shown as dashed lines. H atoms not involved in the crystal structure have been omitted for clarity.

Figure 1d shows the molecular structure of **4**, which contains two crystallographically independent molecules, namely molecules *A* and *B*. The pyrazole rings (N1/N2/C7–C9) in both molecules are almost planar with r.m.s. deviations of 0.0425Å for *A* and 0.0333Å for *B*. In molecule *A*, the pyrazole ring forms dihedral angles of 10.53(10)° with the fluoro-substituted benzene ring (C1A–C6A) and 80.63(10)° with the benzene ring (C10A–C15A). In molecule *B*, the corresponding dihedral angles are 9.78(10) and 79.78(10)°. Bond lengths and angles are within the normal ranges [16]. In the crystal structure of **4** (Figure 5a), molecules *A* and *B* are interlinked via C9B–H9BA•••O1A and C15B–H15B•••O1A hydrogen bonds (Table 2) to form *R*^1^_2_ (6) ring motifs [17] which play a role in stabilizing the crystal structure. These sets of ring motifs are then linked into chains along the [0
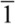

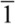
] as shown in Figure 5b via intermolecular C1A–H1AA•••O1B and C1B–H1BA•••O1A hydrogen bonds (Table 2).

## 3. Experimental

### 3.1. Materials and Method

X-ray diffraction studies were carried out using the Bruker SMART Apex II and Apex II Duo CCD diffractometers. Melting points were taken in open capillary tubes and were uncorrected. The purity of the compounds was confirmed by thin layer chromatography using Merck silica gel 60 F_254_ coated aluminium plates. Elemental analyses were carried out by using VARIO EL-III (Elementar Analysensysteme GmBH, Hanau, Germany).

### 3.2. General Procedure for the Synthesis of N-Substituted Pyrazolines

A mixture of substituted chalcone (0.01 mol) and hydrazine hydrate (0.5 mL, 0.01 mol) in 25 mL formic or acetic or propionic acid was refluxed for 8 h. The reaction mixture was cooled and poured into 50 mL ice-cold water. The precipitate was collected by filtration and purified by recrystallization from ethanol. The crystals were grown by the slow evaporation method.

*3-(4-Fluorophenyl)-5-phenyl-4,5-dihydro-1H-pyrazole-1-carbaldehyde* (**1**). Solvent for recrystallization: acetone; Yield: 87%; m.p. 154–157 °C; Analytical data: Found (Cald): C%: 71.60 (71.63); H%: 4.91 (4.88); N%: 10.41 (10.44).

*5-(4-Bromophenyl)-3-(4-fluorophenyl)-4,5-dihydro-1H-pyrazole-1-carbaldehyde* (**2**). Solvent for recrystallization: toluene; Yield: 78%; m.p. 99–101 °C; Analytical data: Found (Cald): C%: 55.32 (55.35); H%: 3.50 (3.48); N%: 8.03 (8.07).

*1-[5-(4-Chlorophenyl)-3-(4-fluorophenyl)-4,5-dihydro-1H-pyrazol-1-yl]ethanone* (**3**). Solvent for recrystallization: ethanol; Yield: 72%; m.p. 110–112 °C; Analytical data: Found (Cald): C%: 64.42 (64.46); H%: 4.49 (4.45); N%: 8.80 (8.84).

*1-[3-(4-Fluorophenyl)-5-phenyl-4,5-dihydro-1H-pyrazol-1-yl]propan-1-one* (**4**). Solvent for recrystallization: acetone; Yield: 67%; m.p. 113–115 °C; Analytical data: Found (Cald): C%: 72.95, (72.97); H%: 5.78, (5.81); N%: 9.45 (9.40).

### 3.3. X-ray Crystallographic Analysis

Selected crystals were mounted on glass fibers and intensity data were collected using either Bruker SMART Apex II or Apex II Duo CCD diffractometer. The data for these compounds were processed with SAINT and corrected for absorption using SADABS. The structures of the compounds were solved by direct method using the program SHELXTL [18], and were refined by full-matrix least squares technique on *F^2^* using anisotropic displacement parameters. The non-hydrogen atoms were refined anisotropically. All the H atoms in these compounds were calculated geometrically with isotropic displacement parameters set to 1.2 (1.5 for methyl groups) times the equivalent isotropic *U* values of the parent carbon atoms. A rotating group model was applied to the methyl groups. Hydrogen bonding interactions are shown in Table 2. CCDC 895315 for (**1**), 895316 for (**2**), 895317 for (**3**) and 895318 for (**4**) contain the supplementary crystallographic data for this paper. These data can be obtained free of charge at http://www.ccdccam.ac.uk/const/retrieving.html or from the Cambridge Crystallographic Data Centre (CCDC), 12 Union Road, Cambridge CB2 1EZ, UK; fax: +44(0)1223-336033 or e-mail: deposit@ccdc.cam.ac.uk.

## 4. Conclusions

The crystal and molecular structures of 3-(4-fluorophenyl)-5-phenyl-4,5-dihydro-1*H*-pyrazole-1-carbaldehyde (**1**), 5-(4-bromophenyl)-3-(4-fluorophenyl)-4,5-dihydro-1*H*-pyrazole-1-carbaldehyde (**2**), 1-[5-(4-chlorophenyl)-3-(4-fluorophenyl)-4,5-dihydro-1*H*-pyrazol-1-yl]ethanone (**3**) and 1-[3-(4-fluorophenyl)-5-phenyl-4,5-dihydro-1*H*-pyrazol-1-yl]propan-1-one (**4**) are reported. These data represent the confirmation of the structures of the four newly reported *N*-substituted pyrazoline compounds.
